# D–A–D-type orange-light emitting thermally activated delayed ﬂuorescence (TADF) materials based on a fluorenone unit: simulation, photoluminescence and electroluminescence studies

**DOI:** 10.3762/bjoc.14.55

**Published:** 2018-03-22

**Authors:** Lin Gan, Xianglong Li, Xinyi Cai, Kunkun Liu, Wei Li, Shi-Jian Su

**Affiliations:** 1 State Key Laboratory of Luminescent Materials and Devices and Institute of Polymer Optoelectronic Materials and Devices, South China University of Technology, Wushan Road 381, Tianhe District, Guangzhou 510640, Guangdong Province, P. R. China

**Keywords:** fluorenone acceptor, orange light emission, organic light-emitting diode (OLED), thermally activated delayed ﬂuorescence

## Abstract

The design of orange-light emitting, thermally activated, delayed ﬂuorescence (TADF) materials is necessary and important for the development and application of organic light-emitting diodes (OLEDs). Herein, two donor–acceptor–donor (D–A–D)-type orange TADF materials based on fluorenone and acridine, namely 2,7-bis(9,9-dimethylacridin-10(9*H*)-yl)-9*H*-fluoren-9-one (27DACRFT, **1**) and 3,6-bis(9,9-dimethylacridin-10(9*H*)-yl)-9*H*-fluoren-9-one (36DACRFT, **2**), were successfully synthetized and characterized. The studies on their structure–property relationship show that the different configurations have a serious effect on the photoluminescence and electroluminescence performance according to the change in singlet–triplet splitting energy (Δ*E*_ST_) and excited state geometry. This indicates that a better configuration design can reduce internal conversion and improve triplet exciton utilization of TADF materials. Importantly, OLEDs based on **2** exhibited a maximum external quantum efficiency of 8.9%, which is higher than the theoretical efficiency of the OLEDs based on conventional fluorescent materials.

## Introduction

Since multilayered OLEDs were first reported by Tang in 1987 [1], organic light-emitting diodes (OLEDs) have been a research focus due to their applications in display devices and general lighting. The efficiency of OLEDs was previously limited by the statistic rule of spin multiplicity. For conventional fluorescent materials, only singlet excitons are involved in electroluminescence, leading to a theoretical maximal internal quantum efﬁciency (IQE_max_) of 25% and a theoretical maximal external quantum efficiency (EQE_max_) of 5%, when assuming the out-coupling efficiency to be 20%. On the other hand, phosphorescent materials could utilize triplet excitons in electroluminescence processes to achieve 100% IQE_max_ [2–3]. However, the utilization of metals like iridium and platinum, which are expensive and nonrenewable, inevitably increase the cost of the final OLEDs. Alternatively, a thermally activated delayed ﬂuorescence (TADF) material is a kind of noble-metal-free fluorescent material able to transform triplet excitons into singlet excitons through reverse intersystem crossing (RISC) to achieve 100% IQE_max_ in theory [4].

On the basis of the previous considerations, for TADF materials, the energy difference (Δ*E*_ST_) between the first singlet excited state (S_1_) and the first triplet excited state (T_1_) must be small enough to enable the RISC process with the activation of environmental thermal energy [5]. To achieve this, electron donors (D) and electron acceptors (A) are introduced into the molecule to form an intramolecular charge transfer (ICT) state with a large twisting angle between the donor and the acceptor to achieve the separation of highest occupied molecular orbital (HOMO) and lowest unoccupied molecular orbital (LUMO) [6], which is the key to reduce the Δ*E*_ST_. Therefore, D–A-type or D–A–D-type molecules are the most classical TADF molecular structures [7].

Although there have been numerous TADF materials synthesized and reported [8–9], to the best of our knowledge, orange and red TADF materials are still rarely reported in comparison with blue and green TADF materials [10–11]. It is difficult to achieve TADF in orange and red fluorescent materials not only because red TADF materials require a strong ICT state, which strongly facilitates nonradiative transition processes, but also because the energy gap law generally results in a low radiative rate constant (*k*_r_) to compete with a large nonradiative rate constant (*k*_nr_) [12]. The increasing nonradiative transition processes and large *k*_nr_ play a role in competition with RISC and radiative transition processes and seriously restrict the development of orange and red TADF materials [5]. Therefore, further attempts and new designs towards orange and red TADF materials are necessary.

**Scheme 1:**
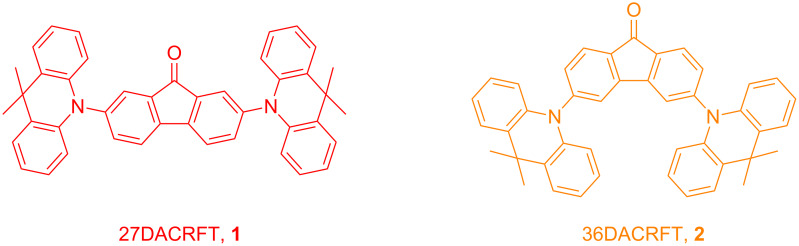
Molecular structures of isomers **1** and **2**.

In this work, we designed and synthetized two novel D–A–D-type orange TADF materials, namely 2,7-bis(9,9-dimethylacridin-10(9*H*)-yl)-9*H*-fluoren-9-one (27DACRFT, **1**) and 3,6-bis(9,9-dimethylacridin-10(9*H*)-yl)-9*H*-fluoren-9-one (36DACRFT, **2**, Scheme 1). The compounds are isomers with different donor–accepter bonding positions, where the fluorenone unit is a strong electron acceptor, which has not been reported in the field of TADF materials before, while acridine, one of the most commonly used donors in TADF materials, has strong electron-donating and hole-transport ability. The combination of the strong acceptor and strong donor can give a narrow energy gap and thus longer wavelength emission. Compounds **1** and **2** were thoroughly characterized by ^1^H NMR, ^13^C NMR and electron ionization (EI) mass spectrometry. Both of them show TADF behavior with orange emission color according to the photoluminescence spectra and time-resolved transient photoluminescence decay measurement. EQEs of 2.9% and 8.9% were achieved for the OLED devices based on **1** and **2**, respectively, which are higher than the theoretical efficiency of the OLEDs based on conventional fluorescent materials.

## Results and Discussion

27DACRFT **1** and 36DACRFT **2** have similar thermal properties according to thermogravimetric analysis (TGA) and differential scanning calorimetry (DSC) measurements. They have high decomposition temperatures (*T*_d_, corresponding to a 5% weight loss) of 361 and 363 °C, respectively. In addition, no glass-transition temperature (*T*_g_) was found according to their DSC curves. Thanks to their amorphous characteristics, the stability of their morphology and chemical composition can be expected during the evaporation processing fabrication of OLEDs.

In order to characterize their electrochemical properties, cyclic voltammetry (CV) measurements were conducted to measure their oxidation potentials (*E*_ox_) and reduction potentials (*E*_red_). Ionization potential (IP) and electron affinity (EA), which approximate to their HOMO and LUMO energy levels, are calculated from *E*_red_ and *E*_ox_. Compounds **1** and **2** have similar HOMO and LUMO energy levels due to the same donor and acceptor in the molecules (Table 1).

**Table 1:** Thermal and electrochemical properties of the investigated compounds **1** and **2**.

Compound	*T*_d_^a^/*T*_g_^b^ (°C)	IP^c^ (eV)	EA^d^ (eV)	*E*_g_^e^ (eV)

**1**	361/N.A.	−3.20	−5.10	1.90
**2**	363/N.A.	−3.15	−5.30	2.15

^a^Decomposition temperature (*T*_d_) at 5 wt % weight loss obtained from TGA measurements; ^b^glass-transition temperature (*T*_g_) obtained by DSC measurements; ^c^ionization potential (IP) calculated from the empirical formula: IP = −(*E*_red_ + 4.4) eV, the cyclic voltammetry was carried out in 0.1 M *n*-Bu_4_NPF_6_ in CH_2_Cl_2_/CH_3_CN 4:1 solution; ^d^electron affinity (EA) calculated from the empirical formula: IP = −(*E*_ox_ + 4.4) eV; ^e^energy gap (*E*_g_) estimated from cyclic voltammetry measurements.

**Figure 1:**
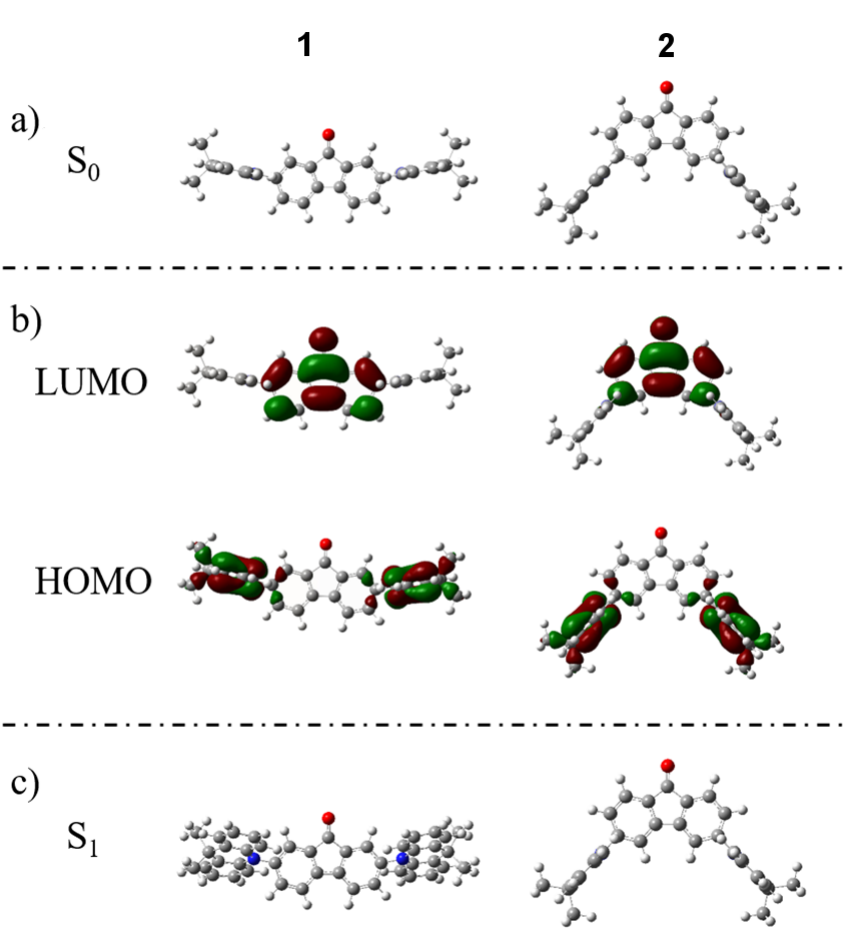
(a) The optimized S_0_ geometries of **1** (left) and **2** (right) on B3LYP/6-31G* level in gas phase; (b) The frontier molecular orbital distributions of **1** and **2**; (c) The optimized S_1_ geometries in TD-DFT on m062x/6-31G* level.

The molecular geometry of **1** and **2** in the ground state and excited state were simulated by density functional theory (DFT) and time-dependent density functional theory (TD-DFT) calculations, respectively. The ground state (S_0_) geometries were optimized on B3LYP/6-31G* level in gas phase, while the lowest triplet excited state (T_1_) energy levels and the singlet excited state (S_1_) geometries of those molecules were optimized by TD-DFT on m062x/6-31G* level based on the optimized ground state geometries. The optimized geometries of S_0_ and S_1_ are shown in Figure 1.

The optimized geometries in S_0_ are shown in Figure 1a, and all the data are summarized in Table 2. Large twisting angles (θ) of 89.33° and 88.80° between the donor units and the accepter units were estimated for compound **1** and **2**, respectively. As shown in Figure 1b, HOMOs and LUMOs are mainly located on the acridine unit and the fluorenone unit, respectively, which contribute to small Δ*E*_ST_. The existence of a very small overlap of HOMOs and LUMOs is advantageous to retain high photoluminescence (PL) quantum yields [13–15]. The calculated Δ*E*_ST_ of **1** and **2** are 0.33 and 0.27 eV, which are small enough to achieve TADF behavior.

**Table 2:** The calculated HOMO, LUMO, twisting angles (θ, θ’), bond lengths (l, l’), Δ*E*_ST_ and dipole moment in gas phase for S_0_ and in solution for S_1_, from DFT and TD-DFT.

Compound	S_0_						S_1_	

	HOMO (eV)	LUMO (eV)	θ (°)	l (Å)	Δ*E*_ST_ (eV)	Dipole moment (D)	θ’ (°)	l’ (Å)

**1**	−5.00	−2.61	89.33	1.433	0.33	3.501	63.74	1.419
**2**	−5.03	−2.61	88.80	1.434	0.27	1.814	89.36	1.434

As shown in Figure 1c, the twisting angle (θ’) of **1** in S_1_ is 63.74°, which is much smaller than its θ in S_0_, meanwhile, the conformation of the acridine units in **1** is also changed in S_1_ as a result of vibrational relaxation and internal conversion (IC), which means the S_0_ geometry of **1** becomes unstable when the molecule is excited and the wave function distribution is changed. The different twisting angles between S_0_ and S_1_ may reduce its PL property according to the energy gap law [16] as vibrational relaxation and intersystem crossing (IC) processes can consume the energy in S_1_, leading to increased nonradiative deactivation [17], reduced PL quantum yield, and thus reduced singlet exciton utilization. On the contrary, the geometry of **2** is hardly changed when excited. Thus, compound **2** shows more potentiality in the application of OLEDs for its better configuration.

**Figure 2:**
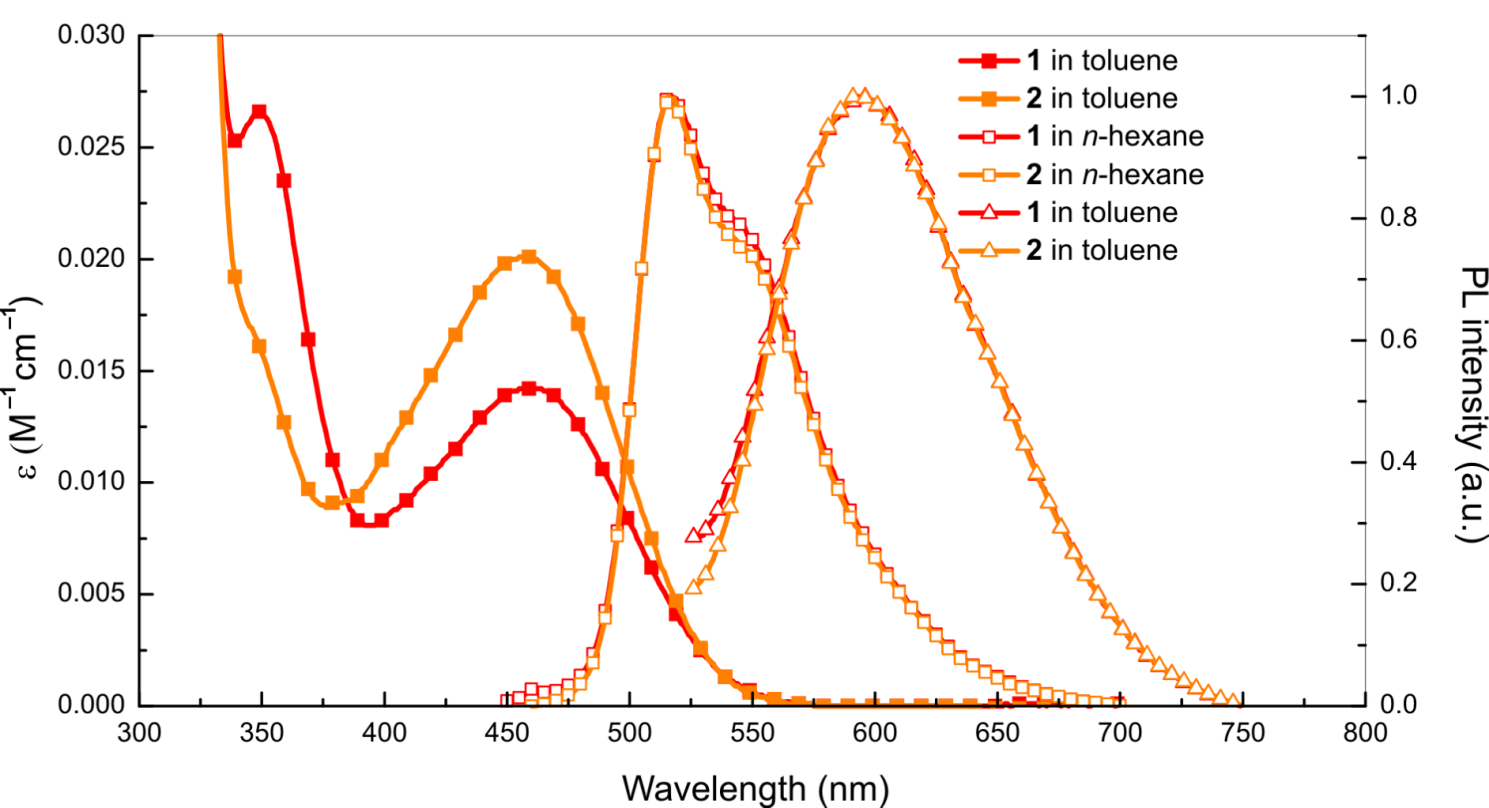
UV–vis (solid point) and photoluminescence (hollow point) spectra of **1** and **2** in dilute solution.

Ultraviolet–visible (UV–vis) absorption and PL spectra in dilute solutions of **1** and **2** (10^−5^ M) are presented in Figure 2. Both compounds **1** and **2** have similar absorption peaks at around 345 and 456 nm. The peaks at around 456 nm result from their ICT states from the donor to the acceptor, while the absorption below 380 nm is caused by their short π-conjugation. It is obvious that **2** has not only a higher oscillator strength (f) than **1** from its transition of charge-transfer states, but also a weaker oscillator strength from its local excited (LE) states. It could be considered that **2** has a better configuration, which is advantageous to intramolecular charge transfer compared with **1**, which coincides with the conclusion from DFT calculation.

The PL spectra of the materials in different solvents were also measured. However, no emission was observed in the dilute solutions of dichloromethane (DCM) and tetrahydrofuran (THF) because vibrational relaxation and internal conversion are promoted to reduce the PL intensity. Both compounds **1** and **2** show almost the same PL spectra in dilute solutions of toluene and *n*-hexane. The photoluminescence spectra of the *n*-hexane solutions show a peak at 517 nm with a shoulder at 545 nm, which can be considered as the radiative transition of ^1^LE states. Noticeably, the charge-transfer process is limited in *n*-hexane because of its lower polarity. Only one peak at 593 nm was observed for the dilute toluene solutions of both molecules with the typical PL spectra from the radiative transition of ICT states, which could be the evidence of the existence of strong ICT states of both molecules. More importantly, both materials achieve orange luminescence in a dilute solution of toluene, which could be attributed to the strong electron-withdrawing ability and excess conjugation length of fluorenone plane compared with conventional benzophenone acceptor [18].

**Figure 3:**
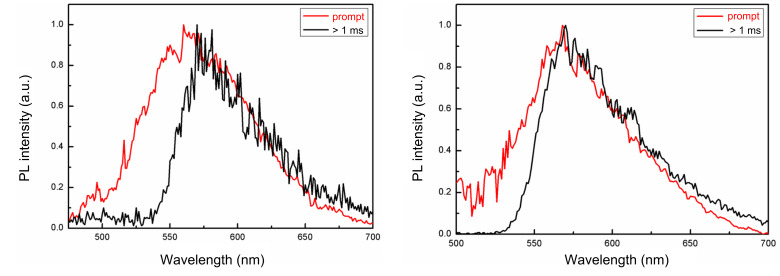
The low temperature photoluminescence spectra of **1** (left) and **2** (right) in toluene at 77 K.

In addition, low temperature photoluminescence (LTPL) spectra of the materials in toluene at 77 K were measured. The energy levels of S_1_ and T_1_ were determined from the onset of the prompt and delayed emission peaks, respectively. As shown in Figure 3, both T_1_ states of the materials could be confirmed as ^3^CT character from their delayed photoluminescence spectra without any well-defined vibronic structure [7]. The Δ*E*_ST_ of **1** and **2** are 0.19 and 0.09 eV, respectively, indicating that compound **2** may have a much more efficient RISC process than **2** [19–20] (Table 3).

**Table 3:** Photophysical properties of the investigated molecules **1** and **2**.

Compound	λ_abs_^a^ (nm)	λ_em_^b^ (nm)	λ_em_^a^ (nm)	λ_em_^c^ (nm)	*E*_g_^d^ (eV)	Φ_PL_^c^ (%)	Δ*E*_ST_^e^ (eV)

**1**	345, 456	517, 545	593	593	2.32	7	0.19
**2**	345, 456	517, 545	593	581	2.32	26	0.09

^a^Ultraviolet–visible absorption spectra and photoluminescence spectra measured in toluene; ^b^photoluminescence (PL) spectra measured in *n*-hexane; ^c^photoluminescence spectra and PL quantum yields measured in doped film 8 wt % in CBP; ^d^energy gap (*E*_g_) calculated from the empirical formula: *E*_g_ = 1240/λ_abs-onset_, where λ_abs-onset_ is the onset of ultraviolet–visible absorption spectra. ^e^Δ*E*_ST_ is calculated from the onset of photoluminescence spectra at 77 K.

To gain a further understanding of the photophysical properties of **1** and **2** in solid state, two doped films in 4,4’-dicarbazolyl-1,1’-biphenyl (CBP) were vacuum co-deposited at a concentration of 8 wt % for photoluminescence quantum yield (PLQY) and time-resolved transient photoluminescence decay measurements. The concentration of the doped films was optimized to ensure complete energy transfer between the host and the guest. PLQY measurements of **1**:CBP and **2**:CBP are 7% and 26%, respectively. The PLQY measurements of the doped films with lower concentration show varying degrees of deviation due to the incomplete energy transfer and the obvious luminescence from CBP (PLQY of **1** and **2** doped in CBP with 1 wt % are 2% and 10%, respectively). As shown in Supporting Information File 1, both PL spectra of the doped films of **1**:CBP and **2**:CBP show red-shift from their PL spectra in *n*-hexane, which could be considered as the influence from aggregation. As mentioned above, **1** and **2** show nearly the same PL spectra in their dilute toluene solution. However, the PL spectrum of **2** is slightly blue-shifted from its PL spectrum in toluene, while **1**:CBP shows alike spectra with **1** in toluene. It could be considered as the solid-state solvation effect [21], as **2** and **1** have different dipole moment of 1.814 D and 3.501 D, respectively from DFT calculation, owing to their different configurations.

**Figure 4:**
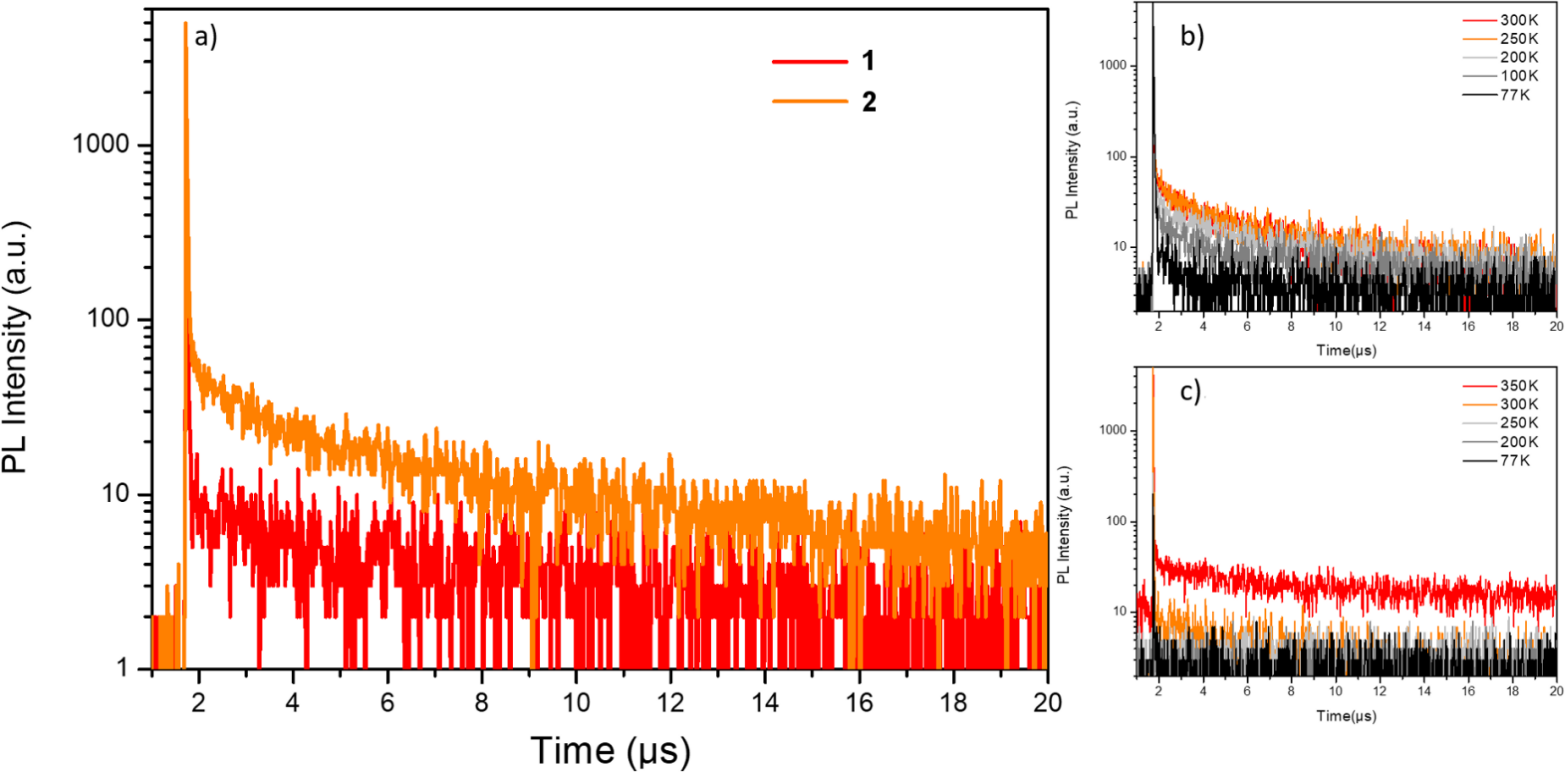
(a) Time-resolved transient photoluminescence decay spectra of the doped films (8 wt % in CBP) measured in N_2_ at 300 K; time-resolved transient photoluminescence decay spectra of (b) **2**:CBP and (c) **1**:CBP measured in N_2_ at different temperatures.

The doped film **2**:CBP shows a typical TADF behavior as shown in Figure 4b, according to the time-resolved transient photoluminescence decay measurement. The proportion of delayed fluorescence increases rapidly with improved temperature from 77 to 250 K and slowly by acceleration of the nonradiative transition rate when the temperature is higher than 250 K. On the other hand, **1**:CBP hardly shows a TADF behavior when the temperature is below 300 K, as shown in Figure 4c.

The signals are characterized by noise rather than delayed fluorescence when the temperature is lower than room temperature due to its low PLQY. Delayed fluorescence can be only observed when the temperature is above 300 K. This could be attributed to the large Δ*E*_ST_ and low PLQY of **1** which requires more energy to achieve RISC process from T_1_ to S_1_. According to the integration and the lifetime of the prompt and delayed components of the time-resolved transient PL decay curves at room temperature, the PLQY of their respective components and rate constant of different kinetic processes were calculated, as shown in Table 4.

**Table 4:** Photophysical properties of the **1** and **2** doped in CBP films (8 wt %) at room temperature.

Compound	Φ	Φ_PF_	Φ_TADF_	τ_PF_ (ns)	τ_TADF_ (μs)	*k*_r_ (10^6^ s^−1^)	*k*_nr_ (10^7^ s^−1^)	*k*_isc_ (10^7^ s^−1^)	*k*_risc_ (10^5^ s^−1^)

**1**	0.07	0.06	0.01	11.6	10.6	5.2	6.91	1.23	1.10
**2**	0.26	0.16	0.10	18.5	4.28	8.6	2.45	2.07	3.81

The rate constants were calculated following Equations 1–4 below [5,7,16].

[1]
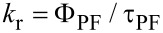


[2]
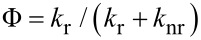


[3]
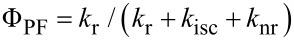


[4]
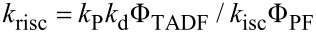


where *k*_r_, *k*_nr_, *k*_isc_, and *k*_risc_ represent the rate constant of radiative, nonradiative, intersystem crossing and reverse intersystem crossing, respectively; Φ, Φ_PF_, Φ_TADF_, τ_PF_ and τ_TADF_ represent the photoluminescence quantum yield, quantum yield of the prompt component, quantum yield of the delayed component, and lifetimes of the prompt and delayed components, respectively. As shown in Table 4, **2** has a significantly larger *k*_nr_ than **2**, which is consistent with the DFT simulation. On the other hand, a much lower *k*_risc_ and longer τ_TADF_ was acquired by **1**:CBP than **2**:CBP, as a result of the blocked reverse intersystem crossing and the large Δ*E*_ST_. Further, the existence of strong IC and vibrational relaxation processes of **1** is proved by its large *k*_nr_ and low PLQY. In contrast, owing to the relatively small Δ*E*_ST_, *k*_risc_ of **2** is higher and τ_TADF_ is relatively shorter than **1**. The short τ_TADF_ not only signifies efficient utilization of singlet excitons, but is also advantageous in reducing the triplet exciton concentration and efficiency roll-off in the OLED devices.

**Figure 5:**
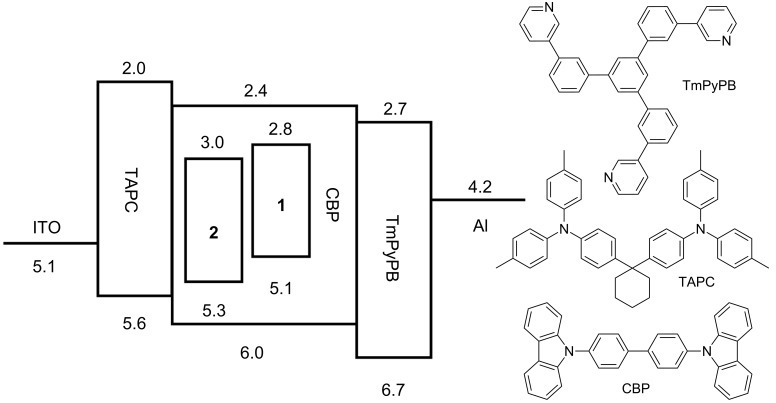
Energy level (eV) diagrams of OLED devices and the chemical structures of the materials utilized for device fabrication.

Finally, electroluminescent properties of **1** and **2** were characterized in a device structure of ITO/TAPC (25 nm)/1 wt % emitter in CBP (35 nm)/TmPyPB (55 nm)/LiF (1 nm)/Al, where 1,1-bis(4-(di-*p*-tolylamino)phenyl)cyclohexane (TAPC), 4,4'-bis(9*H*-carbazol-9-yl)biphenyl (CBP), 1,3,5-tri[(3-pyridyl)-phen-3-yl]benzene (TmPyPB) and LiF play the roles of hole transport layer, host material, electron transport layer and electron injection layer, respectively [22]. The energy level diagrams and the chemical structures of the materials utilized are shown in Figure 5.

**Figure 6:**
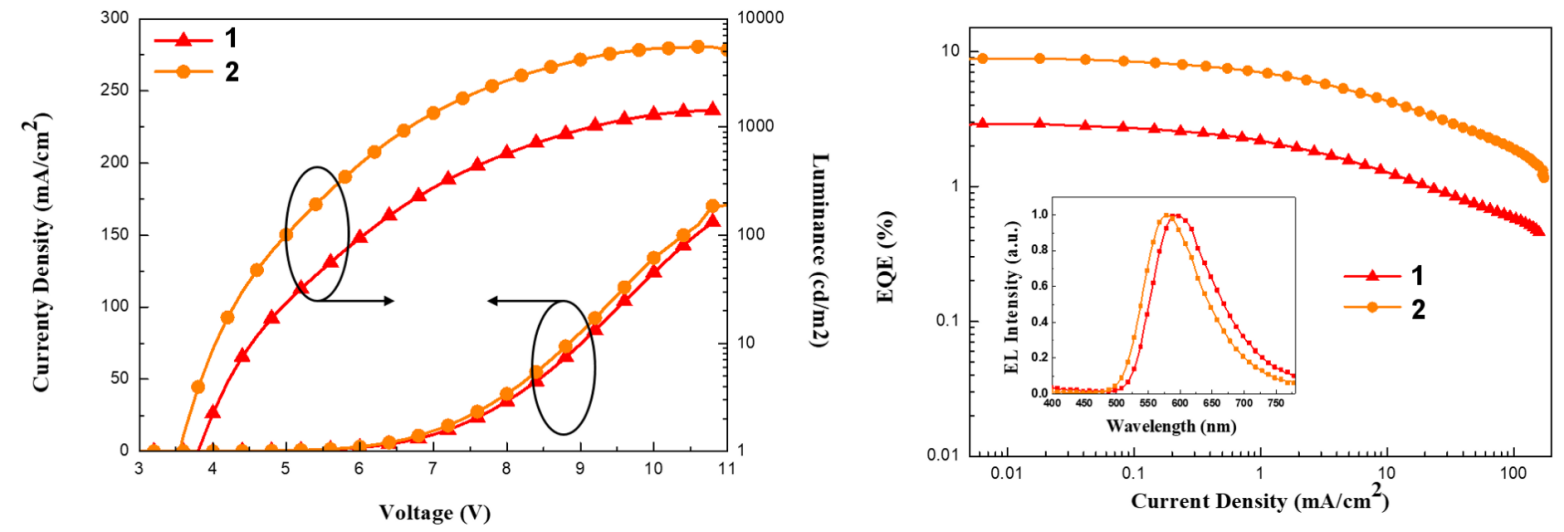
*J*–*V*–*L* (current density–voltage–luminance) (left) and EQE–current density characteristics of the devices (right). Inset: Electroluminescence spectra of the devices at a luminance of 1 cd m^−2^.

TAPC and TmPyPB also play the role of exciton blocking layer at the same time because of their high T_1_ energy level. Carriers will also be trapped by the emitter directly because of the energy level difference between CBP and the emitter, which makes it possible for the OLEDs with such a low emitter concentration to achieve complete energy transfer. The performance of the fabricated devices is summarized in Table 5 while the *J*–*V*–*L* (current density–voltage–luminance) and EQE–current density characteristics of the devices are shown in Figure 6.

**Table 5:** Summary of the device performances of the OLEDs based on **1** and **2**.

Device^a^	*V*_on_^b^ (V)	CE_max_ (cd/A)	PE_max_ (lm/W)	EQE_max_ (%)	at 100 cd/m^2^		at 1000 cd/m^2^	

					V (V)	EQE (%)	V (V)	EQE (%)

**1**	3.8	5.70	4.98	2.93	6.1	1.77	9.2	0.67
**2**	3.6	21.84	19.11	8.92	5.0	7.53	6.7	4.55

^a^The device structure is ITO/TAPC (25 nm)/CBP:**1** or **2** (1 wt %, 35 nm)/TmPyPB (55 nm)/LiF (1 nm)/Al. ^b^At the luminance 1 cd/m^2^.

**Figure 7:**
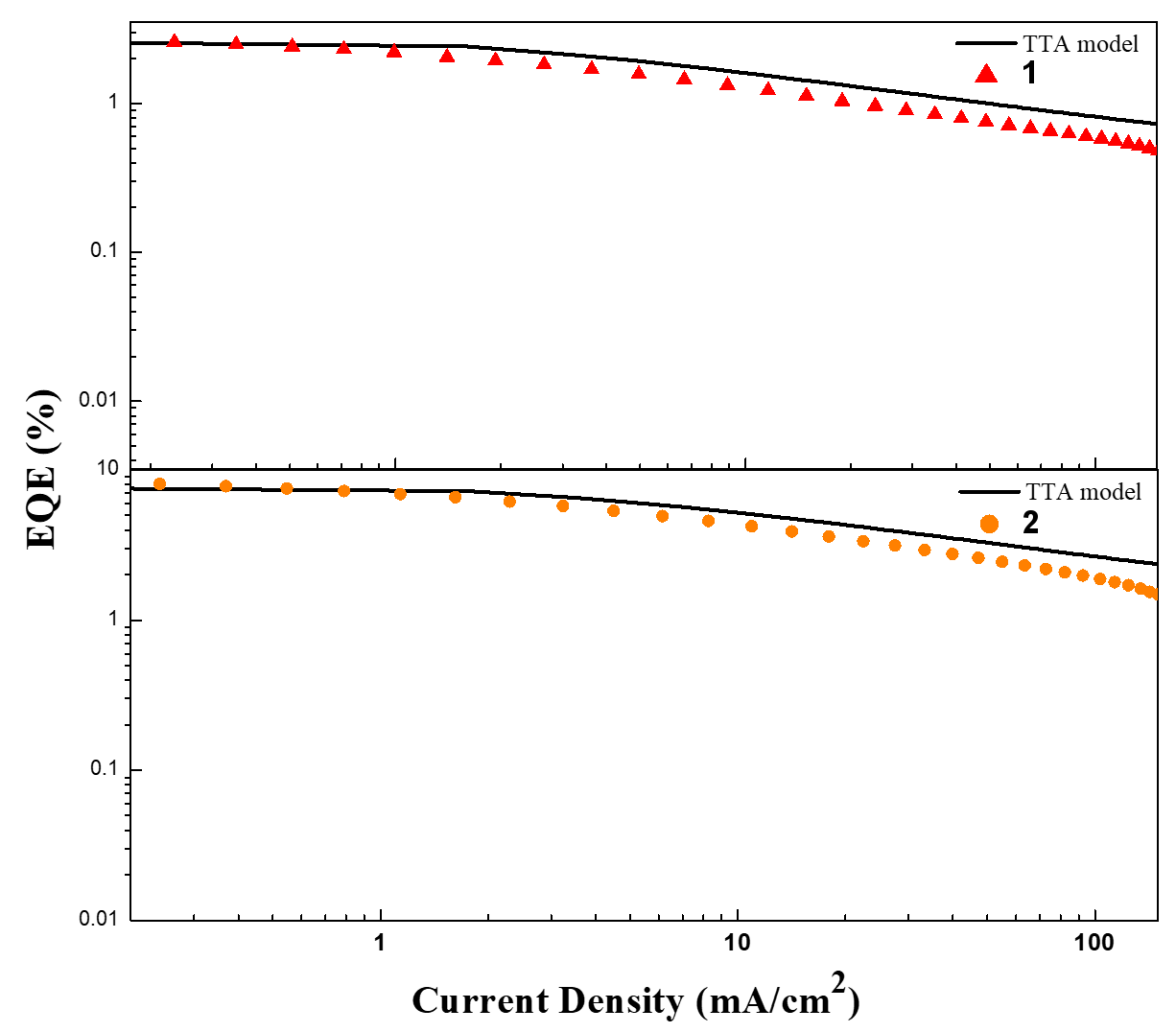
EQE–current density characteristics of the devices based on **1** (top) and **2** (bottom). The solid lines represent the simulated EQE by employing the TTA model.

A significantly higher performance was observed from the device based on **2** with a maximal current efﬁciency (CE_max_) of 21.84 cd/A, maximal power efﬁciency (PE_max_) of 19.11 lm/W and maximal external quantum efficiency (EQE_max_) of 8.92%, which is higher than the theoretical maximal external quantum efficiency of the OLEDs based on conventional fluorescent emitter. Meanwhile, the device based on **1** shows poor performance due to its low PLQY and nonobvious TADF behavior. Moreover, the efficiency roll-off of the device based on **2** was reduced compared with the **1**-based device. The EQE of the **2**-based device is still over half of its EQE_max_ at a brightness of 100 cd/m^2^, while the EQE of **1** at the same brightness is only about 22% of its EQE_max_. According to the previous study, triplet–triplet annihilation (TTA) might be the main cause of efficiency roll-off in the TADF-OLEDs when the triplet exciton concentration increases with brightness and current density [23–24]. The efficiency roll-off caused by the TTA process of TADF-OLEDs could be analyzed by the TTA model using Equation 5 [25–26] below:

[5]
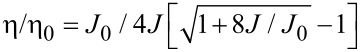


where η_0_ represents the EQE without the influence of TTA, and *J*_0_ represents the current density at the half maximum of the EQE; η and *J* represent the EQE with the influence of TTA and the corresponding current density, respectively. As shown in Figure 7, both devices show good agreement with the TTA model ﬁtted curves at low current density because TTA process is the leading factor to the efficiency roll-off of TADF-OLEDs when the exciton concentration is low. With the increase of exciton concentration, singlet–triplet annihilation (STA), singlet–polaron annihilation (SPA) and triplet–polaron annihilation (TPA) may also have serious impact to the efficiency roll-off, which cause the TTA model ﬁtted curves to deviate from the actual value. The device based on **2** shows a better agreement with the ﬁtted curve in higher current density while the device based on **1** does not. In addition, **2** has a better triplet exciton utilization ability to reduce the efficiency roll-off, which comes to the same conclusion with the analysis of their photophysical properties.

## Conclusion

In summary, two novel D–A–D-type orange-emitting TADF materials, namely 2,7-bis(9,9-dimethylacridin-10(9*H*)-yl)-9*H*-fluoren-9-one (27DACRFT, **1**) and 3,6-bis(9,9-dimethylacridin-10(9*H*)-yl)-9*H*-fluoren-9-one (36DACRFT, **2**), with the fluorenone unit as acceptor and the acridine as donor, were synthetized. Compounds **1** and **2** are isomers but show greatly different performance in terms of both photoluminescence and electroluminescence. It has been shown that the fluorenone unit is a promising acceptor for orange TADF materials, which aids in the design of the TADF behavior and luminescence color of **1** and **2**. Owing to the strong electron-withdrawing ability and extended conjugation length of fluorenone unit, the emission peaks of both materials show obvious red-shifts from other TADF materials based on carbonyl acceptor [27–28]. According to the DFT and TD-DFT simulation and photophysical characterization, **2** shows a smaller singlet–triplet energy difference (Δ*E*_ST_) and a larger radiative rate constant (*k*_r_) to give reduced internal conversion, promoted RISC process, and thus a better triplet exciton utilization ability. Maximum EQE values of 8.9% and 2.9% were achieved for the OLED devices based on **2** and **1**, respectively. Efficiency roll-off, which is considered to be the result of TTA, is also reduced more effectively for the OLEDs based on **2**.

## Experimental

^1^H and ^13^C NMR spectra were measured on a Bruker NMR spectrometer with tetramethylsilane (TMS) as the internal standard. TGA and DSC measurements were performed on a Netzsch TG 209 and a Netzsch DSC 209 under N_2_, respectively. A CHI600D electrochemical work station with a platinum working electrode and a platinum wire counter electrode at a scanning rate of 100 mV s^−1^ against a Ag/Ag^+^ (0.1 M of AgNO_3_ in acetonitrile) reference electrode were utilized for cyclic voltammetry measurements. UV–vis absorption spectra were measured using a HP 8453 spectrophotometer and PL and LTPL spectra were measured with a Jobin-Yvon spectrofluorometer. PLQY spectra were measured on a Hamamatsu absolute PL quantum yield spectrometer C11347. Transient PL spectra were measured with an Edinburgh FL920 fluorescence spectrophotometer. The current density–voltage–luminance characteristics of the OLED devices were measured with a Keithley 2420 and Konica Minolta chromameter CS-200. The EL spectra were measured with a Photo Research PR705 device.

## Supporting Information

File 1 Experimental and additional information.
