# Supplementary material for: D–A–D-type orange-light emitting thermally activated delayed ﬂuorescence (TADF) materials based on a fluorenone unit: simulation, photoluminescence and electroluminescence studies
Source: Beilstein J Org Chem. 2018 Mar 22;14:672–81. doi: 10.3762/bjoc.14.55 (PMC5870154; doi:10.3762/bjoc.14.55)
Supplement: File 1 — Experimental and additional information. [file Beilstein_J_Org_Chem-14-672-s001.pdf]

Supporting Information

for

D–A–D-type orange-light emitting thermally activated delayed fluorescence (TADF) materials based on a fluorenone unit: simulation, photoluminescence and electroluminescence studies

Lin Gan, Xianglong Li, Xinyi Cai, Kunkun Liu, Wei Li and Shi-Jian Su*

Address: State Key Laboratory of Luminescent Materials and Devices and Institute of Polymer Optoelectronic Materials and Devices, South China University of Technology, Wushan Road 381, Tianhe District, Guangzhou 510640, Guangdong Province, P. R. China

Email: Shi-Jian Su - mssjsu@scut.edu.cn

*Corresponding author

Experimental and additional information

Contents

Synthesis of materials S2

Cyclic voltammetry curves S3

TGA and DSC curves S4

Normalized PL spectra of doped films S4

References S5

Materials: Solvents and reagents were purchased from commercial sources and used as received. Raw materials of 2,7-dibromo-9-fluorenone, 3,6-dibromo-9-fluorenone and 9,9-dimethyl-9,10-dihydroacridine were synthesized according to literature procedures [1,2]. 27DACRFT (**1**) and 36DACRFT (**2**) were further purified by recrystallization and repeated temperature gradient vacuum sublimation.

Synthesis of 27DACRFT (1): 2,7-Dibromo-9-fluorenone (0.676 g, 2 mmol), 9,9-dimethyl-9,10-dihydroacridine (1.046 g, 5 mmol), sodium *tert*-butoxide (0.48 g, 5 mmol) and palladium acetate (45 mg, 0.2 mmol) were dissolved in toluene (180 mL) and stirred under an argon (Ar) flow in a 250 mL three-necked flask for 30 minutes. Then, tris(1,1-dimethylethyl)phosphine (1.0 M solution in toluene, 0.4 mL) was dropped into the mixture, followed by heating under reflux for 72 hours. The formed precipitate was extracted with dichloromethane (DCM) and washed with deionized water after cooling to room temperature. The organic layer was separated and further purified by chromatography on silica gel to afford the title compound as orange powder (0.7 g, 58%) after evaporation of the solvent. ^{1}H NMR (500 MHz, $CDCl_3$): δ 7.96 (d, *J* = 7.8 Hz, 1H), 7.46 (dd, *J* = 7.8, 1.6 Hz, 3H), 7.35 (dd, *J* = 7.8, 1.7 Hz, 1H), 7.00 (dddd, *J* = 16.3, 14.9, 7.3, 1.5 Hz, 4H), 6.48 (dd, *J* = 8.1, 1.3 Hz, 2H), 1.66 (s, 6H). ^{13}C NMR (126 MHz, $CDCl_3$): δ 191.52 (s), 148.06 (s), 146.52 (s), 140.18 (s), 133.31 (s), 131.37 (s), 126.74 (s), 126.49 (s), 125.41 (s), 122.56 (s), 121.49 (s), 114.85 (s), 36.16 (s), 30.94 (s). EI-MS (electron ionization mass spectrometry, m/z): calcd for $C_{43}H_{34}N_2O$ 594.76; found 595.7 [M +].

Synthesis of 36DACRFT (2): 36DACRFT (**2**) was synthesized in a similar way as described for 27DACRFT (**1**) starting from 3,6-dibromo-9-fluorenone instead of 2,7-dibromo-9-fluorenone (0.676 g, 2 mmol). The precipitate was extracted with dichloromethane (DCM) and washed with deionized water after cooling to room temperature. The organic layer was separated and further purified by chromatography on silica gel to afford the title compound as orange powder (0.6 g, 50%) after evaporation of the solvent. ^{1}H NMR (500 MHz, $CDCl_3$): δ 7.95 (d, *J* = 7.8 Hz, 1H), 7.46 (dd, *J* = 7.7, 1.6 Hz, 3H), 7.35 (dd, *J* = 7.8, 1.7 Hz, 1H), 6.99 (dtd, *J* = 25.2, 7.3, 1.4 Hz, 4H), 6.48 (dd, *J* = 8.1, 1.3 Hz, 2H), 1.66 (s, 6H). ^{13}C NMR (126 MHz, $CDCl_3$): δ 148.06 (s), 146.52 (s), 140.18 (s), 133.31 (s), 131.37 (s), 126.73 (s), 126.49 (s), 125.41 (s), 122.56 (s), 121.49 (s), 114.85

(s), 36.16 (s), 30.94 (s). EI-MS (electron ionization mass spectrometry, m/z): calcd for $C_{43}H_{34}N_2O$ 594.76; found 595.6 [M +].

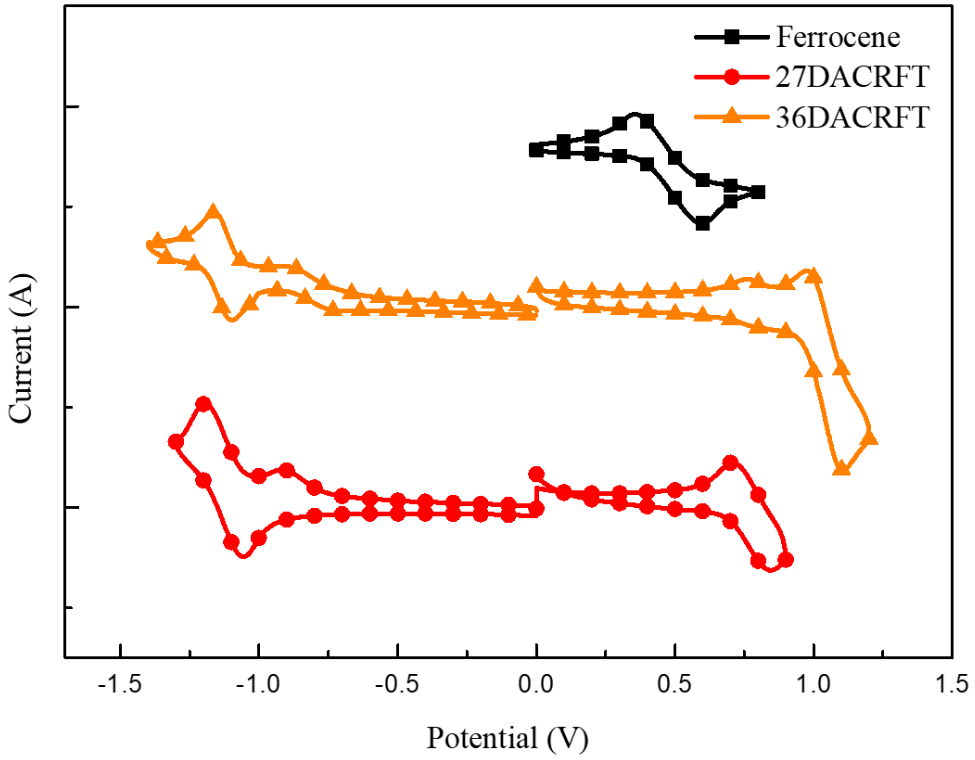

Figure S1: Cyclic voltammetry curves of 27DACRFT (**1**), 36DACRFT (**2**) and ferrocene. The oxidation potential (E_{ox}) and reduction potential (E_{red}) were determined from the onset of the oxidation and reduction curves.

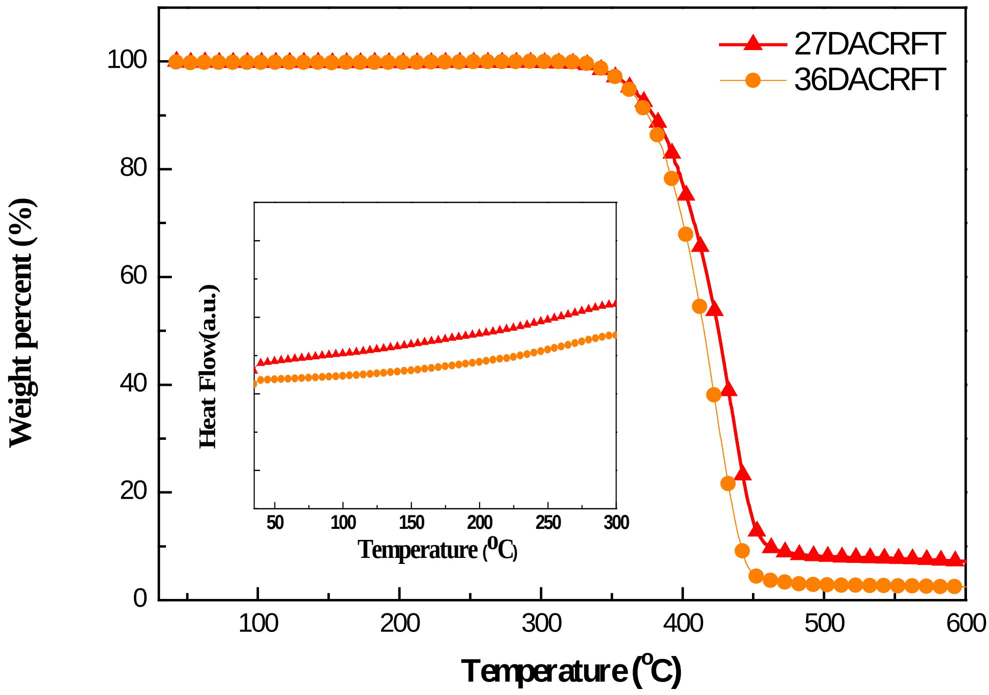

Figure S2: TGA and DSC (inset) curves of 27DACRFT (**1**) and 36DACRFT (**2**).

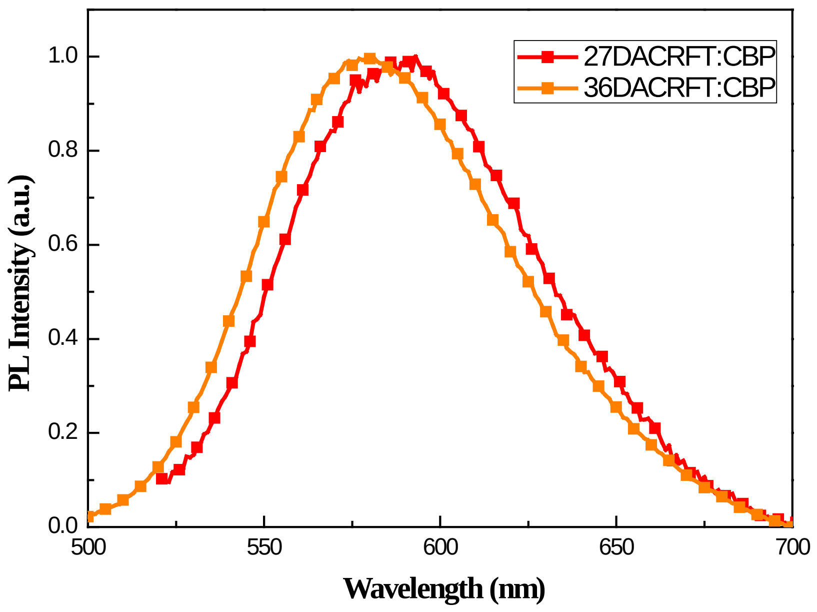

Figure S3. Normalized PL spectra of the doped films of 27DACRFT:CBP and 36DACRFT:CBP in a concentration of 6wt%.

References

1. Zhang, Q., Li, B., Huang, S., Nomura, H., Tanaka, H., and Adachi, C., Nat. Photon., 2014. **8**: 326-332.
2. Wu, Z., Xiong, Y., Zou, J., Wang, L., Liu, J., Chen, Q., Yang, W., Peng, J., and Cao, Y., Adv. Mater., 2008. **20**: 2359-2364.
